# Chronic Rigidity of the Spine

**Published:** 1899-12-30

**Authors:** 


### CHRONIC RIGIDITY OF THE SPINE.

Every now and again one comes across a case in which there is marked bowing of the spine, with considerable fixity, but without any obvious sign of bone disease. Sometimes, no doubt, this is a deformity caused partly by occupation and partly by rheumatism, as in the case of coachmen, ploughmen, and agricultural labourers, whose work involves long-continued stooping, and much exposure to cold and wet. Again, such a condition may arise as part of a general rheumatic state, or of general arthritis deformans. Lately, considerable attention has been paid to these and similar conditions, and various attempts have been made to classify them, and to ascertain their origin. Following the description given by various authors, cases of chronic stiffness of the vertebral column, other than those mentioned above, may be broadly divided into two classes, which have lately been carefully described by Dr. Charles L. Dana.[1] The first is the condition referred to by Strümpell[2] in 1884, when in his chapter on arthritis deformans, he says: "A remarkable, and, as it seems to us, unique disorder may be mentioned in passing. It leads to complete ankylosis of the entire spinal column and the hip-joints, so that the head, trunk, and thighs are firmly united and stiffened, while all the other joints retain their normal mobility." This condition has been further investigated by Marie,[3] who has called it spondylosis rhizomelia. In this the malady begins usually in the hip-joints, and progresses steadily upwards, affecting the joints of spine and even the shoulder. Its marked sign is rigidity; but there is also kyphosis, although this may not be great. The patient does not have much pain, and does not suffer from the intercostal neuralgias or shooting pains which occur in the second class of cases. The second type is that described by Bechterew. In these the trouble begins in the spine primarily, and is accompanied with a progressive stiffness of and arching of the back, which may become very great. The patient suffers much from intercostal pains, while the joints of the hip and shoulder are not very much, if at all involved. According to Marie, there is a history of heredity, and often of trauma. In regard to the pathology of these conditions the first question is whether the spondylosis rhizomelia of Marie is not simply a form of arthritis deformans. The chief points which, it is claimed, distinguish spondylosis are (1) the fact that it produces directly an ankylosis without any previous inflammatory swelling, exudation and proliferation, and that it produces this ankylosis directly, and not as the secondary result of a deformity; (2) that it begins in the vertebral joints, and extends thence to the hips and other large joints; (3) that the smaller joints are never affected, or only rarely in one or two cases; (4) the internal organs and nervous system remain normal; (5) the clinical picture is one of great uniformity; (6) there is no previous history of rheumatism or gout, and no evidence of arthritis deformans in any other part of the skeleton; and (7) the affection occurs mostly in men in about middle life. As for the other, or Bechterew type, it seems to be a secondary affection, and may be caused by a chronic meningitis or may in some cases be a form of rheumatoid arthritis. Taking the facts of the case altogether, however, Dr. Dana says that the case for this being a specifically new malady does not appear to be made out, and he is inclined to believe that, after all, spondylosis rhizomelia is but a form of arthritis deformans, allied to that trouble in etiology, symptomatology, and progress, while some of Bechterew's cases are probably instances of the same disease, and others are due to syphilitic meningitis.

[1] New York Med. News, Nov. 25. [2] Text Book of Medicine. [3] Revue de Medicine, April, 1898.

